# Supplementary material for: Conditional Pten knockout in parvalbumin- or somatostatin-positive neurons sufficiently leads to autism-related behavioral phenotypes
Source: Mol Brain. 2021 Jan 27;14:24. doi: 10.1186/s13041-021-00731-8 (PMC7839207; doi:10.1186/s13041-021-00731-8)
Supplement: Supplementary file 1 — Additional file 1: Figure S1. PTEN expression in parvalbumin positive neurons of PV-Pten mice. Figure S2. PTEN expression in somatostatin positive neurons of Sst-Pten mice. Figure S3. No pre-existing side preference during the habituation session in modified three-chamber social tests for both PV-Pten and Sst-Pten mice. Figure S4. Cre expression in PV- or Sst-neurons did not change most behavioral performance. Figure S5. No significant correlation between behavioral performance and ages in PV-Pten-WT mice. Figure S6. No significant correlation between behavioral performance and ages in Sst-Pten-WT mice. Figure S7. No sex effect in open-field test and marble burying test was observed in PV-Pten or Sst-Pten mice. Figure S8. No sex effect in social test, rotarod test and EPM test was observed in PV-Pten or Sst-Pten mice. [file 13041_2021_731_MOESM1_ESM.pdf]

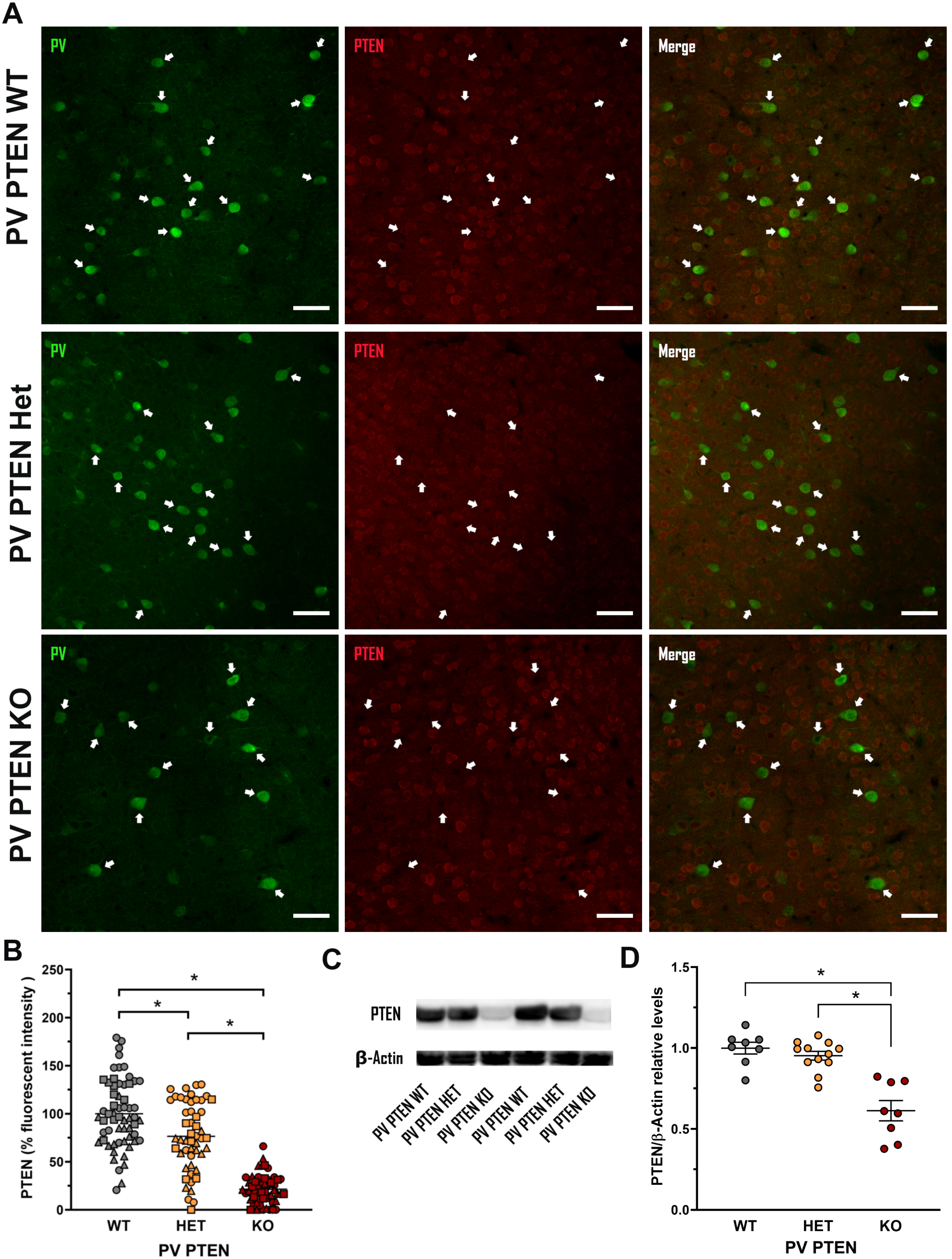
A
PV PTEN WT
PV PTEN Het
PV PTEN KO
PV
PTEN
Merge
B
PTEN (% fluorescent intensity)
250
200
150
100
50
0
*
*
*
WT
HET
KO
PV PTEN
C
PTEN
β-Actin
PV PTEN WT
PV PTEN HET
PV PTEN KO
PV PTEN WT
PV PTEN HET
PV PTEN KO
D
PTEN/β-Actin relative levels
1.5
1.0
0.5
0.0
*
*
WT
HET
KO
PV PTEN

Fig. S1

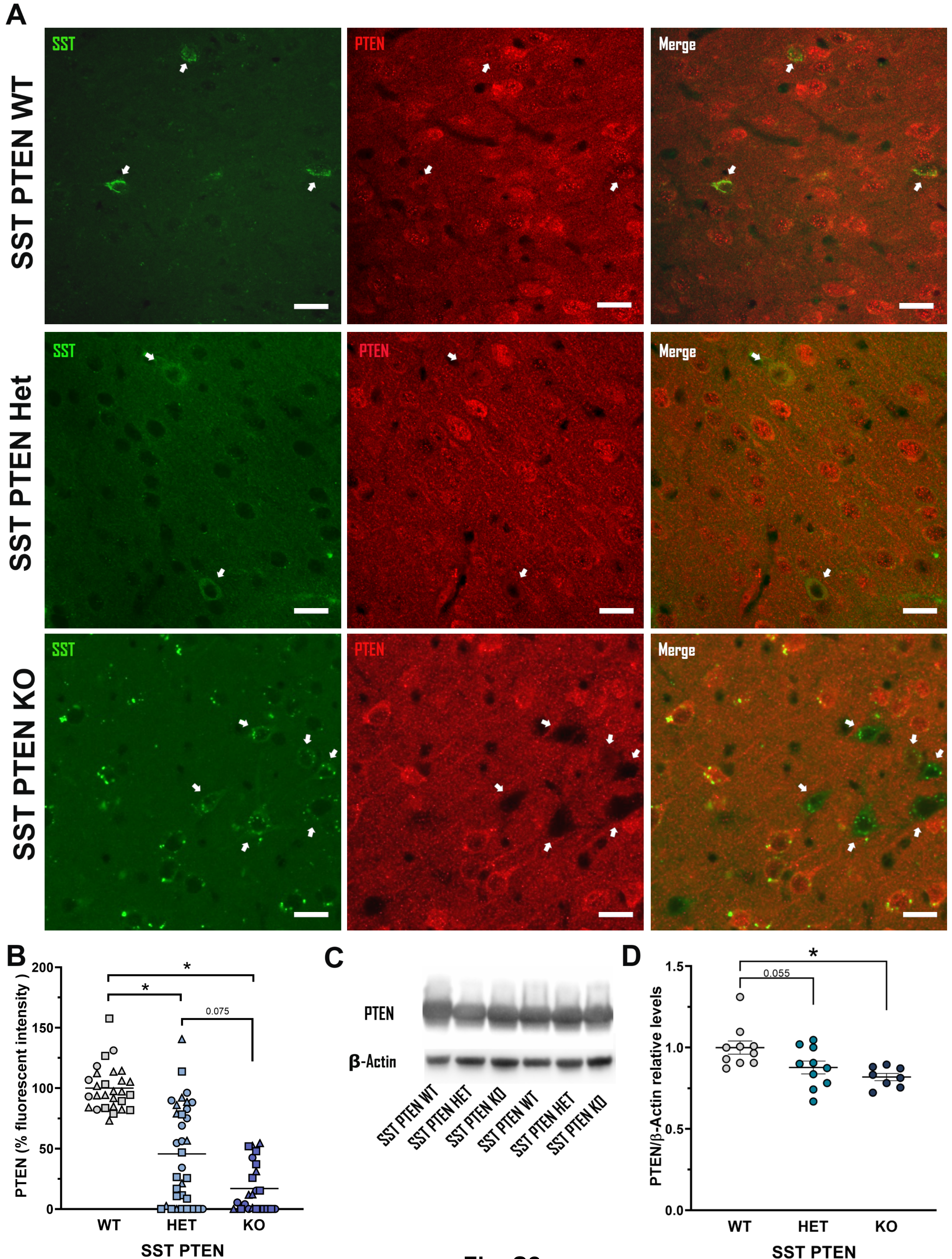
A
SST PTEN WT
SST PTEN Het
SST PTEN KO
SST
PTEN
Merge
B
PTEN (% fluorescent intensity)
200
150
100
50
0
*
*
0.075
WT
HET
KO
SST PTEN
C
PTEN
β-Actin
SST PTEN WT
SST PTEN HET
SST PTEN KO
SST PTEN WT
SST PTEN HET
SST PTEN KO
D
PTEN/β-Actin relative levels
1.5
1.0
0.5
0.0
*
0.055
WT
HET
KO
SST PTEN

Fig. S2

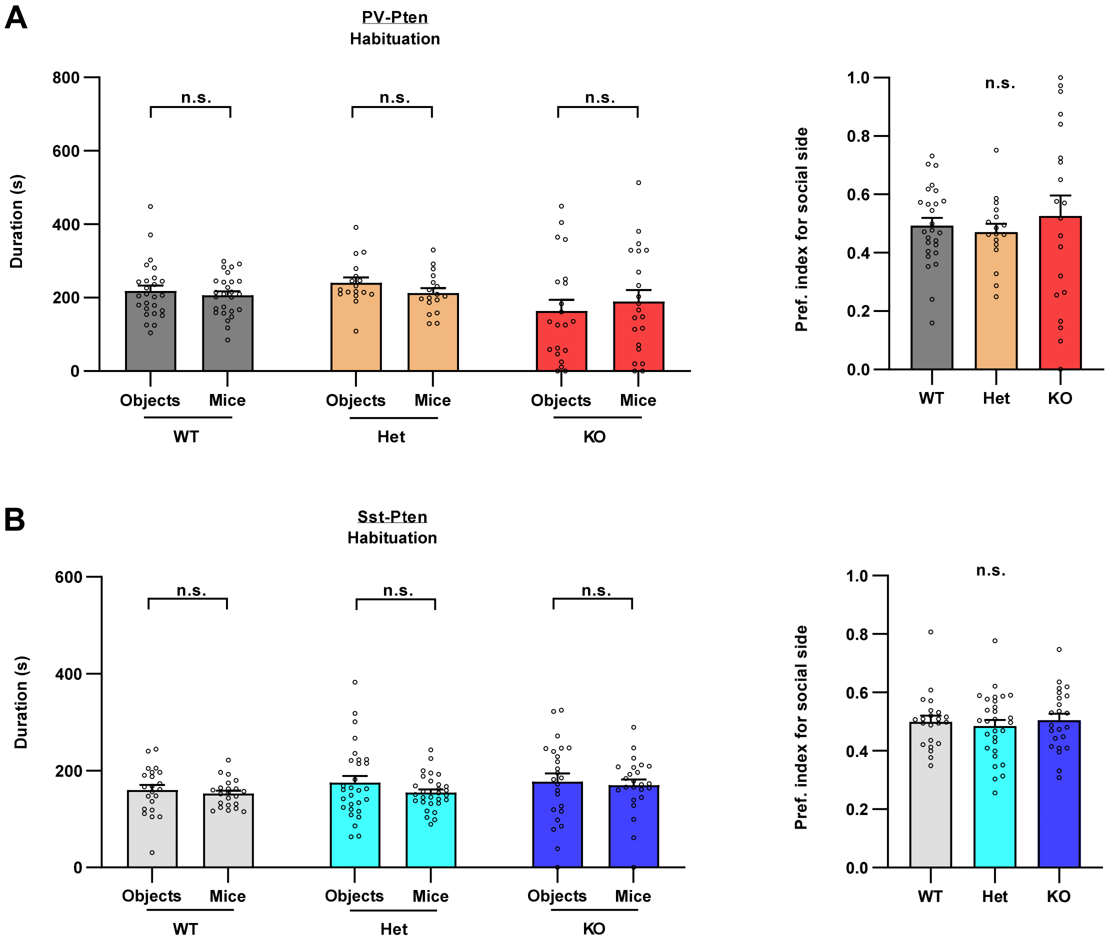
A
PV-Pten
Habituation
Duration (s)
800
600
400
200
0
n.s.
n.s.
n.s.
Objects
Mice
Objects
Mice
Objects
Mice
WT
Het
KO
Pref. index for social side
1.0
0.8
0.6
0.4
0.2
0.0
n.s.
WT
Het
KO
B
Sst-Pten
Habituation
Duration (s)
600
400
200
0
n.s.
n.s.
n.s.
Objects
Mice
Objects
Mice
Objects
Mice
WT
Het
KO
Pref. index for social side
1.0
0.8
0.6
0.4
0.2
0.0
n.s.
WT
Het
KO

Fig. S3

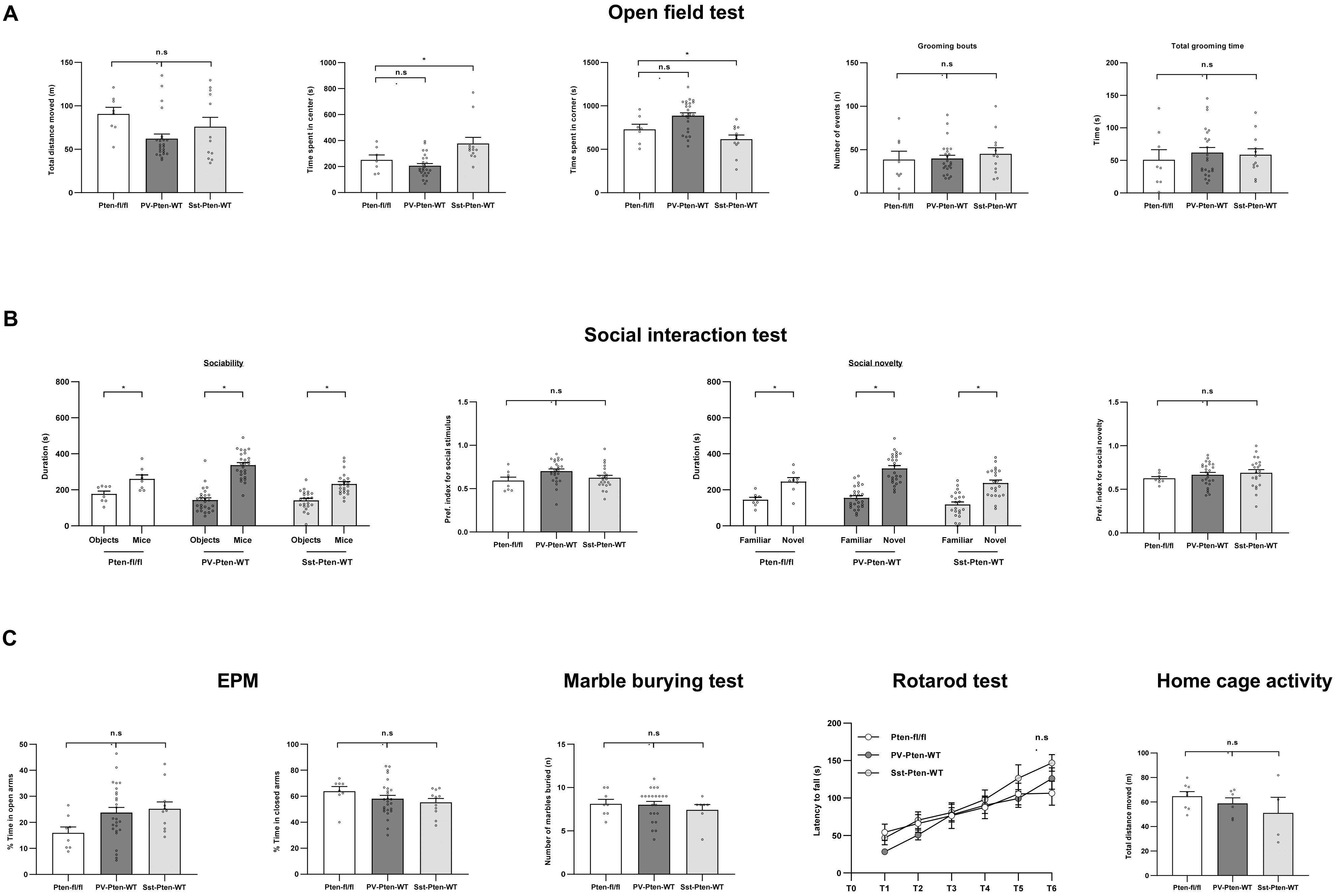
A
Open field test
Total distance moved (m)
Time spent in center (s)
Time spent in corner (s)
Grooming bouts
Number of events (n)
Total grooming time
Time (s)
n.s
*
Pten-fl/fl
PV-Pten-WT
Sst-Pten-WT
B
Social interaction test
Sociability
Duration (s)
Objects
Mice
Pref. index for social stimulus
Social novelty
Familiar
Novel
Pref. index for social novelty
C
EPM
% Time in open arms
% Time in closed arms
Marble burying test
Number of marbles buried (n)
Rotarod test
Latency to fall (s)
T0
T1
T2
T3
T4
T5
T6
Home cage activity
Total distance moved (m)

Fig. S4

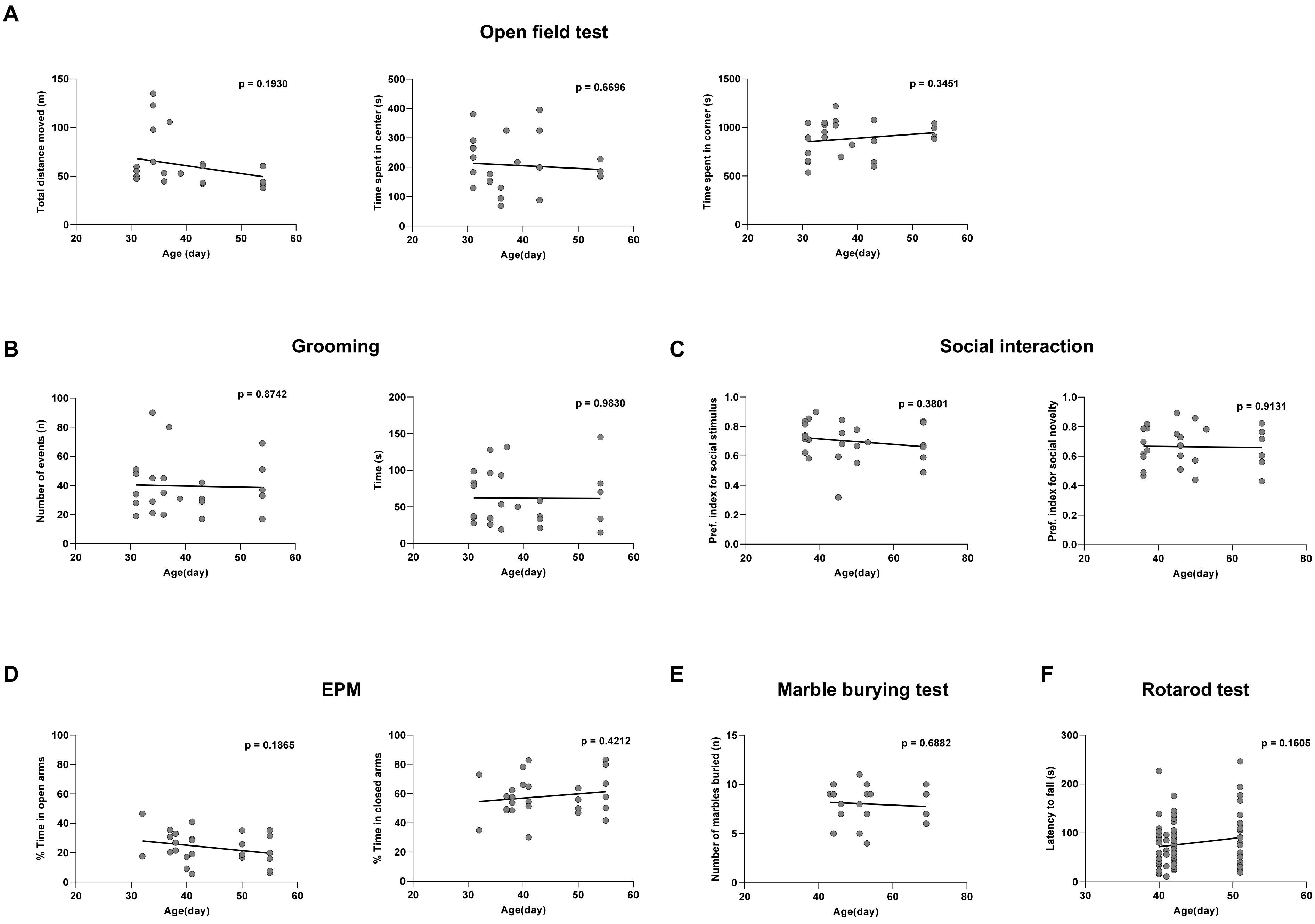

A
Open field test
Total distance moved (m)
Age (day)
p = 0.1930
Time spent in center (s)
Age(day)
p = 0.6696
Time spent in corner (s)
Age(day)
p = 0.3451
B
Grooming
Number of events (n)
Age(day)
p = 0.8742
Time (s)
Age(day)
p = 0.9830
C
Social interaction
Pref. index for social stimulus
Age(day)
p = 0.3801
Pref. index for social novelty
Age(day)
p = 0.9131
D
EPM
% Time in open arms
Age(day)
p = 0.1865
% Time in closed arms
Age(day)
p = 0.4212
E
Marble burying test
Number of marbles buried (n)
Age(day)
p = 0.6882
F
Rotarod test
Latency to fall (s)
Age(day)
p = 0.1605

Fig. S5

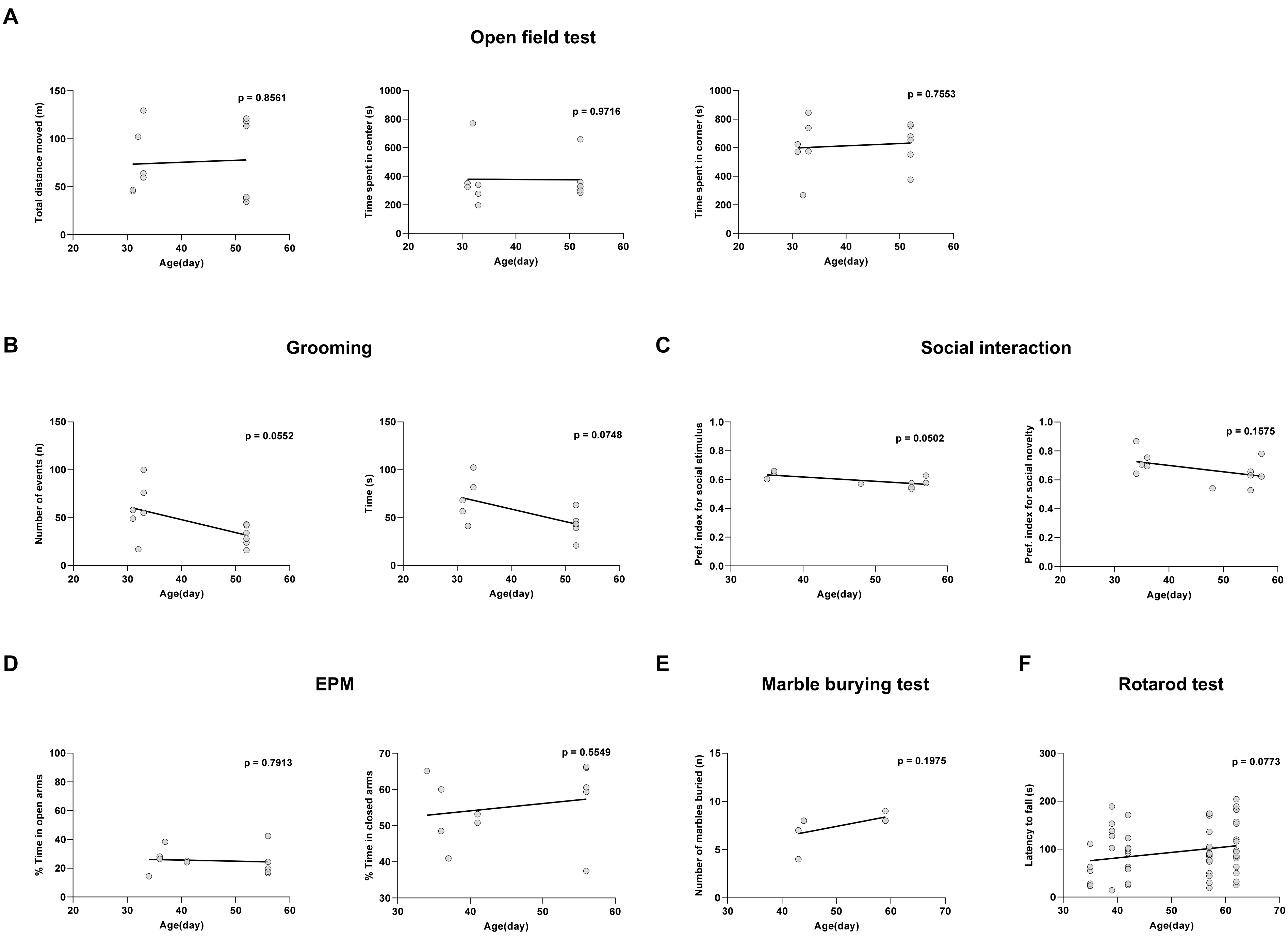
A
Open field test
Total distance moved (m)
p = 0.8561
Time spent in center (s)
p = 0.9716
Time spent in corner (s)
p = 0.7553
Age(day)
B
Grooming
Number of events (n)
p = 0.0552
Time (s)
p = 0.0748
C
Social interaction
Pref. index for social stimulus
p = 0.0502
Pref. index for social novelty
p = 0.1575
D
EPM
% Time in open arms
p = 0.7913
% Time in closed arms
p = 0.5549
E
Marble burying test
Number of marbles buried (n)
p = 0.1975
F
Rotarod test
Latency to fall (s)
p = 0.0773

Fig. S6

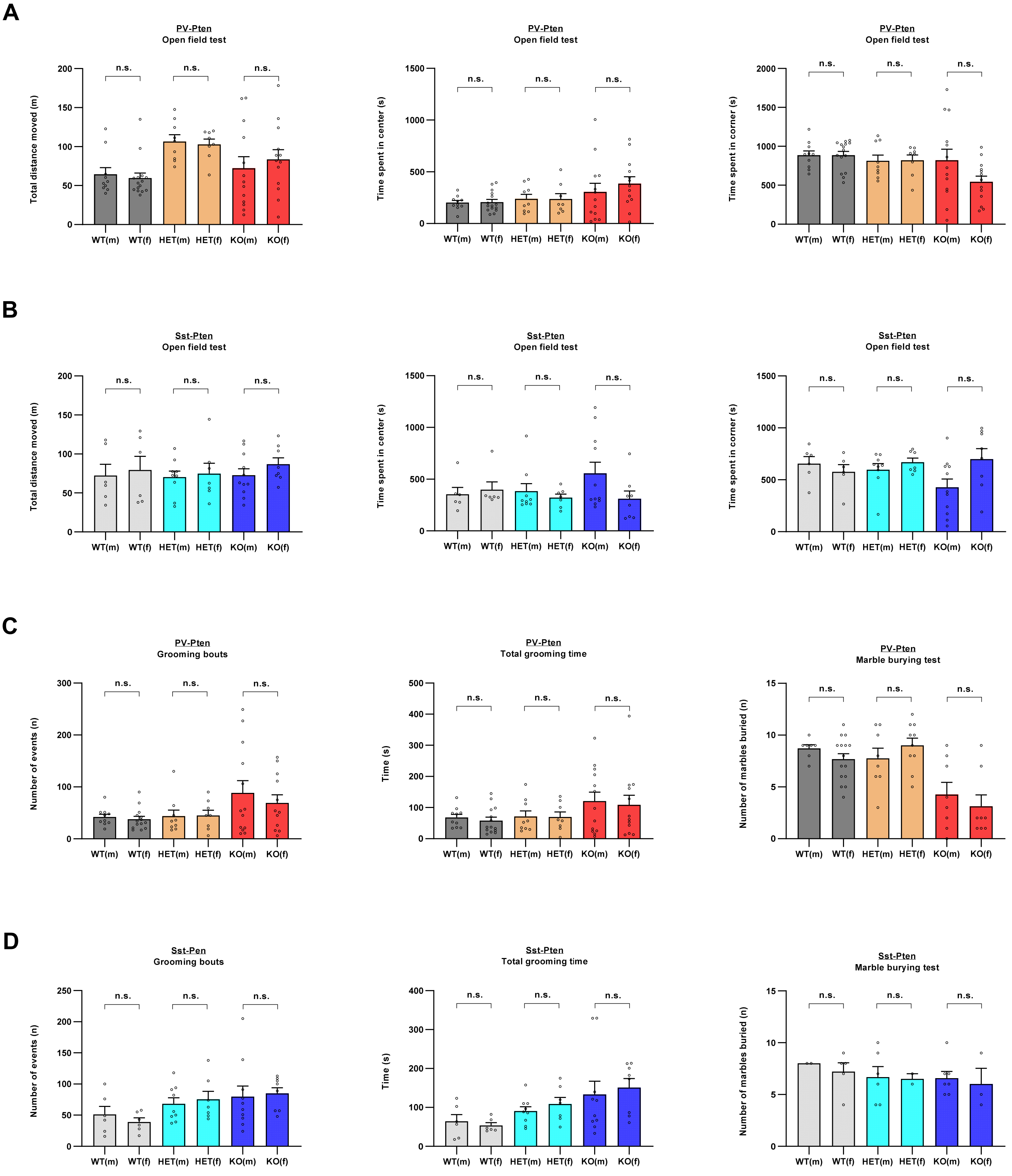
A
PV-Pten
Open field test
Total distance moved (m)
Time spent in center (s)
Time spent in corner (s)
n.s.
WT(m)
WT(f)
HET(m)
HET(f)
KO(m)
KO(f)
B
Sst-Pten
Open field test
Total distance moved (m)
Time spent in center (s)
Time spent in corner (s)
C
PV-Pten
Grooming bouts
Number of events (n)
PV-Pten
Total grooming time
Time (s)
PV-Pten
Marble burying test
Number of marbles buried (n)
D
Sst-Pen
Grooming bouts
Number of events (n)
Sst-Pten
Total grooming time
Time (s)
Sst-Pten
Marble burying test
Number of marbles buried (n)

Fig. S7

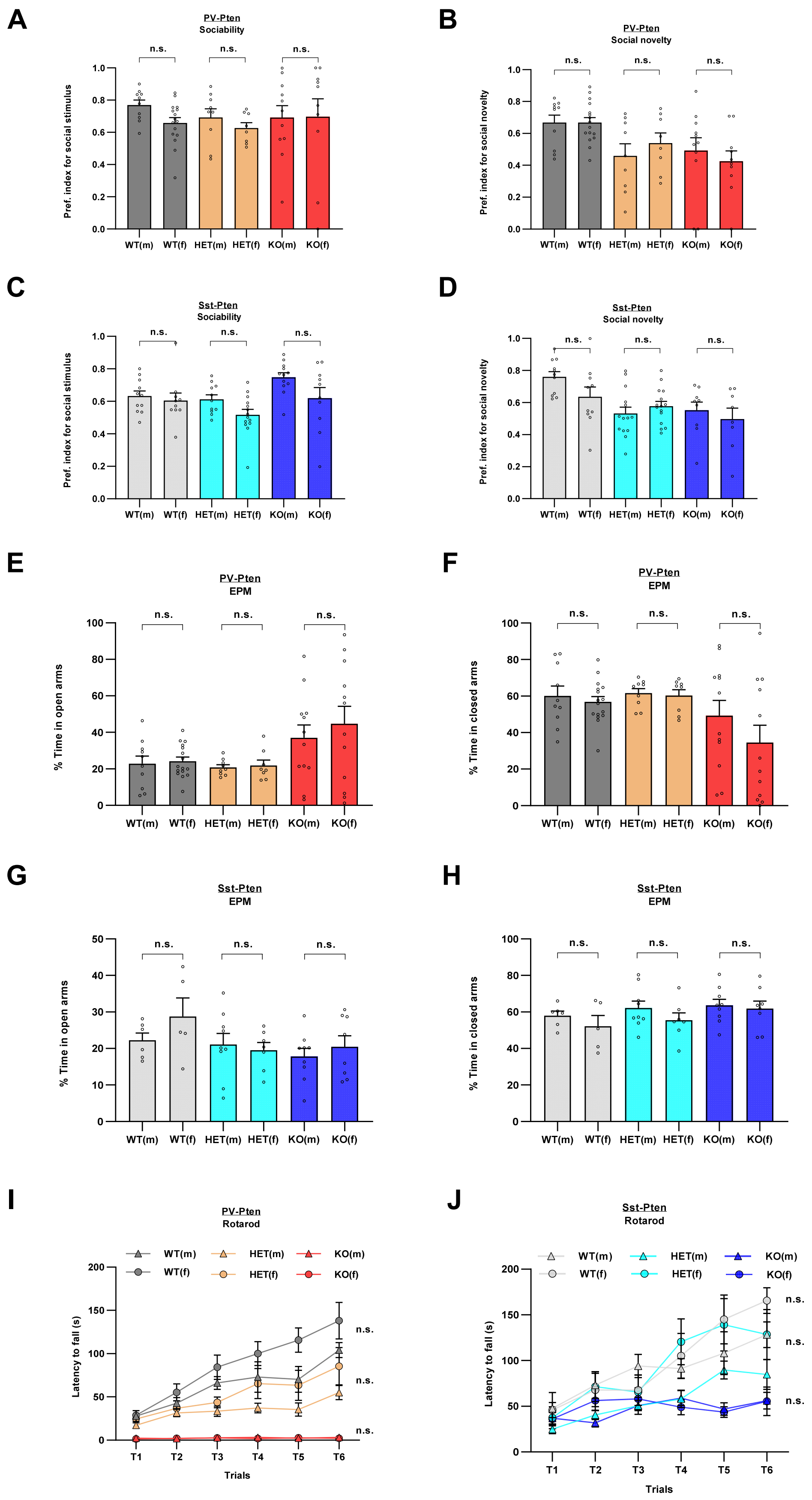

A
PV-Pten
Sociability
Pref. index for social stimulus
B
PV-Pten
Social novelty
Pref. index for social novelty
C
Sst-Pten
Sociability
D
Sst-Pten
Social novelty
E
PV-Pten
EPM
% Time in open arms
F
PV-Pten
EPM
% Time in closed arms
G
Sst-Pten
EPM
H
Sst-Pten
EPM
I
PV-Pten
Rotarod
Latency to fall (s)
Trials
J
Sst-Pten
Rotarod
WT(m)
WT(f)
HET(m)
HET(f)
KO(m)
KO(f)
n.s.
T1 T2 T3 T4 T5 T6

Fig. S8
